# Editorial: Multi-target drug discovery and design for complex health disorders

**DOI:** 10.3389/fphar.2025.1633600

**Published:** 2025-06-13

**Authors:** Farid A. Badria, Barbara De Filippis, Mohammed Abu El-Magd, Mostafa M. Elbadawi, Abdelrahman Hamdi, Abdullah A. Elgazar

**Affiliations:** ^1^ Department of Pharmacognosy, Faculty of Pharmacy, Mansoura University, Mansoura, Egypt; ^2^ Department of Pharmacy, “G. d’Annunzio” University, Chieti, Italy; ^3^ Department of Anatomy, Faculty of Veterinary Medicine, Kafrelsheikh University, Kafrelsheikh, Egypt; ^4^ Department of Pharmaceutical Chemistry, Faculty of Pharmacy, Kafrelsheikh University, Kafrelsheikh, Egypt; ^5^ Department of Pharmaceutical Organic Chemistry, Faculty of Pharmacy, Mansoura University, Mansoura, Egypt; ^6^ Department of Pharmacognosy, Faculty of Pharmacy, Kafrelsheikh University, Kafrelsheikh, Egypt

**Keywords:** multitarget, drug discovery, drug design, health disorders, natural products, *in silico* drug discovery

Multi-target drug discovery represents a pivotal advancement in addressing complex health conditions in the context of global resource limitations. Diseases such as cancer, neurodegenerative disorders, and diabetes are often characterized by multifactorial etiologies, necessitating innovative therapeutic strategies (Mesiti et al., 2019). Drugs that act on a single target have frequently proven impractical and insufficient for managing these complex conditions. In contrast, multi-targeting approaches that leverage computational chemistry, artificial intelligence (AI), and modern drug discovery techniques offer more practical and cost-effective solutions (Alcaro et al., 2019).

Simultaneous modulation of multiple biological targets enhances drug efficacy while reducing side effects and toxicity. The integration of diverse disciplines—from molecular biology to medicinal chemistry—provides a deeper understanding of complex disorders and drives the development of more effective treatments (Cerchia and Lavecchia, 2023). This paradigm shift not only paves the way to improving patient outcomes but also contributes to sustainable healthcare by potentially reducing the number of medications required for treatment.

Thus, the multi-target drug discovery approach emerges as a key strategy in advancing personalized medicine and efficiently addressing global health challenges. A key aspect of this paradigm is distinguishing “multi-target drugs” from related concepts such as “multi-activity drugs.” Multi-target drugs are specifically designed to engage multiple predefined therapeutic targets within a disease pathway, thereby enhancing efficacy and minimizing toxicity. Conversely, multi-activity drugs exhibit a broad pharmacological profile that can affect multiple systems nonspecifically. Clear definitions and contextual understanding are critical for advancing this field (de Sena Murteira Pinheiro et al., 2024).

Natural products (NPs) are a rich source of multi-activity drugs that exert their effects by binding to a wide array of targets. Numerous studies have demonstrated the intrinsic ability of NPs to modulate multiple targets. For instance, a single compound may target several key enzymes involved in pathways of specific or related disorders (Maruca et al., 2019). Various strategies have been employed to enhance the targeting capabilities of natural and synthetic products, including structural optimization through chemical synthesis. This approach has improved the activity of several NPs (Ibrahim et al., 2019).

The exploration and development of multi-targeting agents have consistently shown improved therapeutic outcomes (Shaaban et al., 2025). These agents modulate multiple pathways simultaneously, resulting in enhanced efficacy and reduced side effects—critical factors in the management of complex health disorders. The advantages of multi-target drug discovery include enhanced therapeutic efficacy, reduced polypharmacy, and improved patient outcomes. However, significant challenges remain, including the complexity of preclinical validation, high development costs, and the potential for adverse interactions between therapeutic targets. Developing reliable computational models and experimental systems to predict multi-target effects remains a substantial hurdle (Mukaidaisi et al., 2024).

This Research Topic stems from our firsthand experience with the crucial role of multi-targeting strategies in developing effective pharmacological agents. Drawing from real-world cases, we have witnessed how this approach significantly enhances therapeutic efficacy. The seven studies presented here illuminate the evolving landscape of multi-target drug discovery by showcasing the integration of computational, biological, and natural product-based methods. Together, these studies demonstrate innovative strategies for addressing complex diseases, reinforcing our conviction in the power of multifaceted approaches.

The first article on phenotypic drug discovery (PDD) by Garaci et al. provides an in-depth analysis of thymosin alpha-1 as a model for investigating host-microbe interactions. By adopting a PDD approach, this study breaks away from traditional single-target methodologies, offering a fresh perspective on drug discovery. The potential of PDD lies in its ability to uncover therapeutic effects without predefined molecular targets, thus facilitating the discovery of compounds that address diseases driven by multifactorial mechanisms.

A particularly impactful study on ankylosing spondylitis (AS) by Zhang et al. leverages genetic association, Mendelian randomization, and protein-protein interaction analyses to identify key proteins, such as MAPK14, as potential therapeutic targets. The integration of genetic data with molecular docking exemplifies the synergy between computational and biological methods. This approach is a prime example of how data-driven models can guide the identification of novel therapeutic targets, reaffirming the importance of hybrid approaches in tackling complex diseases like AS.

The exploration of the effects of trimetazidine in rheumatoid arthritis (RA) by Omran et al. is another noteworthy contribution. By modulating the miRNA128a and TLR4 pathways, this study unveils the multi-targeted anti-inflammatory potential of the compound. This work integrates both *in vivo* and *in silico* methodologies, illustrating how hybrid techniques can be used to understand the broader, multi-dimensional effects of a single compound. These types of approaches are becoming increasingly critical in unraveling the complex pathogenesis of autoimmune diseases such as RA.

An exciting contribution comes from the study by Ashour on propolis, a natural antioxidant, in mitigating diabetes-induced testicular injury. Due to its effect on oxidative stress and DNA damage repair, propolis is emerging as a promising therapeutic agent. This research underscores the growing interest in natural products as multi-target therapies, particularly in the context of metabolic disorders. The potential of natural compounds in drug discovery is a recurring theme in this Research Topic, demonstrating their relevance in addressing diseases with multifaceted etiologies.

The investigation into the traditional herbal formulation YinChen WuLing Powder (YCWLP) for non-alcoholic steatohepatitis (NASH) by Yuan et al. highlights the role of traditional medicine in modern drug discovery. By targeting the SHP2/PI3K/NLRP3 pathway, this study combines network pharmacology with molecular docking to elucidate the multi-target mechanisms of YCWLP. It serves as a model for how traditional formulations can be scientifically validated and incorporated into contemporary therapeutic strategies for complex conditions such as NASH.

The review on gut microecology and uric acid metabolism by Wang et al. further supports the therapeutic potential of natural products, particularly in managing metabolic disorders such as hyperuricemia. By focusing on the regulation of gut microbiota, the study explores an emerging frontier in drug discovery—one that integrates natural compounds and microbiome research to address multifactorial diseases.

Finally, the development of SAL0114 by Xiao et al. showcases the potential of informed multi-target drug design for treating depression. This novel deuterated dextromethorphan-bupropion combination exemplifies how targeting multiple disease-associated pathways can enhance both efficacy and safety. By simultaneously improving therapeutic activity, metabolic stability, and safety profiles, SAL0114 demonstrates the promise of this approach in addressing complex neurological disorders.

This Research Topic reflects the growing need for multi-target therapeutic strategies and highlights the integration of advanced computational methods, genetic data, and natural products in the pursuit of novel drug discoveries. The contributions in this Research Topic showcase innovative approaches to addressing disease complexities, ultimately driving the field of multi-target drug discovery forward. Nonetheless, the development of reliable computational models and experimental systems, particularly those integrating omics data and artificial intelligence, remains essential for accurately predicting multitarget effects. These tools are critical for addressing key challenges in the field, including the complexity of preclinical validation, high development costs, and the risk of adverse interactions between therapeutic targets.
